# Postural behavior of howler monkeys (*Alouatta palliata*, *A. macconnelli*, and *A. caraya*) during sleep: an assessment across the genus range

**DOI:** 10.5194/pb-7-25-2020

**Published:** 2020-10-01

**Authors:** Bernardo Urbani, Dionisios Youlatos, Martín M. Kowalewski

**Affiliations:** 1 Centro de Antropología, Instituto Venezolano de Investigaciones Científicas, Apartado 66.755, Caracas 1061-A, Venezuela; 2 Department of Zoology, School of Biology, Aristotle University of Thessaloniki, 54124 Thessaloniki, Greece; 3 Estación Biológica Corrientes, Museo Argentino de Ciencias Naturales “Bernardino Rivadavia”, CONICET, Ruta Prov. 8, Km 7, 3400, San Cayetano, Corrientes, Argentina

## Abstract

Sleep is the longest and most continuous behavioral phase in the 24 h cycle of mammals. However, selection of postures, substrates, and tree parts during sleep has not been adequately explored, as well as their evolutionary consequences. The present study investigates postural behavior, substrate, and tree part use during sleep in three howler species (*A. palliata*, *A. macconnelli*, and *A. caraya*) in Nicaragua, French Guiana, and Argentina. All three species were consistent in the use of a crouched ball-like sit-in posture on large, horizontal, unramified, or bifurcated substrates, and in avoiding the periphery of tree crowns. The regularities of these sleeping patterns are very likely functionally associated with protection from potential predators and extreme weather conditions, biomechanical stability, thermoregulation, and enhancement of the digestive process of hard-to-decompose plant material.

## 1 Introduction

Primates, as a primarily arboreal radiation, tend to exhibit a remarkable diversity of locomotor modes and postures, which are fundamental for utilizing the 3-dimensional and complex environment of forest canopies (Prost, 1965; Grand, 1984; Cant, 1992; Bergeson, 1996). Although postural data would provide information about the adaptive significance and evolution of the biological roles of different morphological complexes, postural modes in relation to substrate use have been often neglected in many ecological studies (Garber and Pruetz, 1995; Hunt et al., 1996; Youlatos, 2004).

Locomotor modes and postures constitute major adaptations to exploit econiches and have been strongly diversified in most major primate radiations (Youlatos, 2004; Garber, 2010). Thorough examination of factors at multiple levels, from ecomorphological traits to habitat structure, should help elucidate the ecological pressures that have shaped specific adaptive radiations and, consequently, the evolution of locomotor and postural diversity. In effect, postures, defined as the dynamic maintenance of body stability, are essential in foraging, digestion, social interactions, energy conservation, thermoregulation, and resting/sleeping (Hunt et al., 1996). The latter constitutes the longest and most continuous behavioral phase throughout the 24 h cycle of any primate (Anderson, 1984, 1998, 2000; Fruth and McGrew, 1998; Matsuda et al., 2009; Prasetyo et al., 2009; Wada and Tokida, 1981). Sleeping postures may represent important adaptations for homeostasis and survival and ultimately contribute to fitness (Anderson, 1998, 2000).

Given the lack of data on sleeping postural behavior, it remains largely unexplored which postures primates adopt, which substrates they use, and where they position themselves in tree crowns during sleep. These variables may have important evolutionary implications for survival since selection of stable substrates promotes overall body stability and energy conservation (Rose, 1974), selection of covered areas in the canopy protects from potential predators and extreme weather conditions (Anderson, 1998; Wahungo, 2001), and certain postures contribute to thermoregulation (Anderson, 2000) or digestive processes (Urbani and Bosque, 2007). To test these links, interspecific comparisons are very useful for investigating relevant ecological pressures, associated evolutionary patterns, and taxon-specific constraints and strategies. More particularly, comparisons between closely related taxa, like congeners under different ecological conditions, can be very instructive. In this context, a good example would be howler monkeys (*Alouatta*), a widely distributed and behaviorally flexible neotropical primate genus (Eisenberg et al., 1972; Kowalewski and Zunino, 2004; Kowalewski, 2007; Di Fiore et al., 2011; Cortés-Ortiz et al., 2015; Kowalewski et al., 2015).

Thus, the present study aims to examine the use of postures, substrate types, sizes and inclinations, and tree crown parts during sleep in mantled howlers (*A. palliata*), Guianan red howlers (*A. macconnelli*), and black-and-gold howlers (*A. caraya*). *Alouatta palliata* is found in the northern distributional limits of the genus (Central and north-eastern South America), are sexually dimorphic (males 4.5–9.8 kg, females 3.1–7.6 kg), exploit dry to humid forests, are mainly folivorous, and exhibit high levels of intra-sexual tolerance with large groups containing as many as 4–12 adult males (Garber et al., 1999; Di Fiore et al., 2011). *Alouatta macconnelli* is widespread along the northern parts of South America, display sexual dimorphism (males 7.2–8.0 kg, females 5 kg), exploit mainly humid primary and secondary rain forests, are folivorous–frugivorous, and form small groups that rarely contain more than two adult males (Crockett and Eisenberg, 1987; Crockett and Janson, 2000; Di Fiore et al., 2011). Finally, *Alouatta caraya* is the species with the southernmost range (northern Argentina), is sexually dimorphic (males 4.0–9.6 kg, females 3.8–5.4 kg) and dichromatic, exploits seasonal forests, is folivorous–frugivorous, and may form small uni-male (1 male, 1–5 females) or multi-male–multi-female groups that contain 4–6 males and 4–8 females (Crocket and Eisenberg, 1987; Neville et al., 1988; Rumiz, 1990; Zunino et al., 2001; Kowalewski and Zunino, 2004; Di Fiore and Campbell, 2010). The analysis of their behavior, substrate, and tree part used will help to evaluate the ways posture, sleeping time, physiology, and digestion are intertwined and to test an ecophysiological hypothesis.

The main goal of this research is to explore the potential ecological and adaptive roles of postures during sleep among different species of howler monkeys. Thus, the following questions are addressed:

- Does sleeping postural behavior differ among howler species at various sites?
- What is the potential role of given postures in howler biology?
- Do patterns of postures during sleep reflect an ecological adaptation or are they related to phylogenetic constrains?

## 2 Methods

### 2.1 Study sites

**Figure 1:**
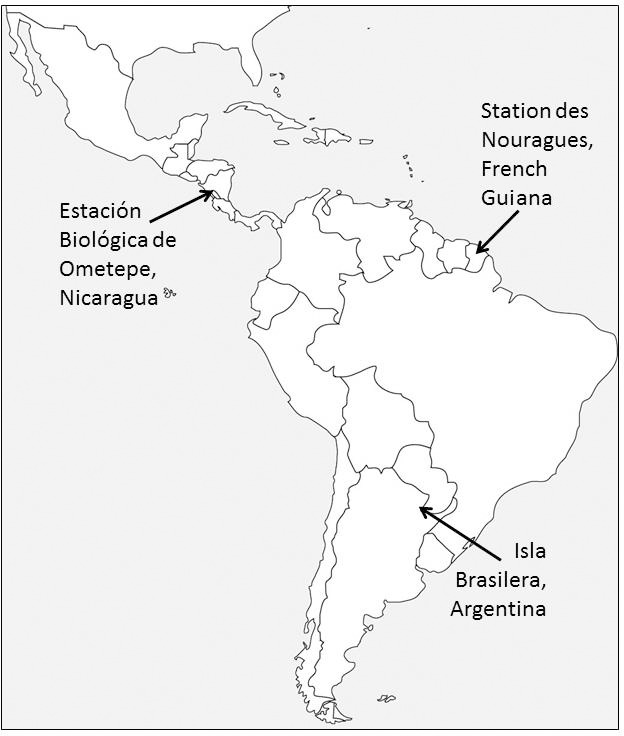
Distribution of the field sites in Nicaragua, French Guiana, and Argentina.

The study was carried out at three different sites covering most of the distributional range of the genus *Alouatta*: Nicaragua in Central America in the northern part of its range, French Guiana in northern South America in the middle of the range, and Argentina in the southwestern limit of South America (Fig. 1).

For the purpose of this research, we collected data on two groups of each studied species. Mantled howler monkey (*Alouatta palliata*) groups were observed in two forests near the Estación Biológica de Ometepe, Nicaragua. In 2003–2004, the group was composed of five adult males, eight adult females, and three young individuals, and in 2006 by four adult males, nine adult females, and four young individuals. The station is located on the southwestern side of the Volcán Maderas in Isla de Ometepe (11${}^{\circ }$40${}^{\prime }$30 N, 85${}^{\circ }$50${}^{\prime }$ W). The vegetation of this site is a Mesoamerican (Pacific) semi-deciduous dry forest with the following dominant tree families: Moraceae, Burseraceae, and Fabaceae (Salas-Estrada, 1993; Garber et al., 1999). The rainy season ranges from June to November, while the dry season lasts from December. The mean annual rainfall ranges from 1200 to 1600 mm (Salas-Estrada, 1993).

Guianan red howler monkeys (*Alouatta macconnelli*) were studied at the Station des Nouragues, French Guiana (4${}^{\circ }$05${}^{\prime }$ N, 52${}^{\circ }$40${}^{\prime }$ W). During fieldwork we observed two groups, composed of a total of two adult males and five adult females. This site is a tropical lowland evergreen primary forest within a matrix of Guianan inselberg geological formations (Grimaldi and Riéra, 2001). The major tree families at the site are Burseraceae, Chrysobalanaceae, Sapotaceae, and Lecythidaceae (Youlatos, 1998; Poncy et al., 2001). During most of the year, the site is affected by an extended wet season. The dry season is concentrated between September and November, and a short dry period occurs in March. The mean temperature is 26 ${}^{\circ }$C, and the annual rainfall average is 3000 mm (Boyé et al., 1979; Youlatos, 1998; Grimaldi and Riéra, 2001).

Two groups of black-and-gold howlers (*Alouatta caraya*) were followed at the site of Isla Brasilera in the northern Chaco region of Argentina (27${}^{\circ }$10${}^{\prime }$ S, 58${}^{\circ }$38${}^{\prime }$ W). The first group was composed of one adult male, one subadult male, three adult females, two subadult females, three juveniles, and two infants; the second group was composed of two adult males, three adult females, one subadult female, two juveniles, and one infant. The site is a flooded forest near the confluence of the Paraná–Paraguay rivers, and tree vegetation is dominated by Lauraceae and Moraceae (Kowalewski, 2007). This forest has periodic floods that produce a continuous deposition of sediments and nutrients, favoring vegetation with a high rate of recovery and resistance to inundation (Popolizio, 1977; Franceschi and Lewis, 1979; Eskuche and Fontana, 1996). At this site, there are four defined seasons (winter: June–August, spring: September–November, summer: December–March, fall: April–May; Kowalewski and Zunino, 2004; Kowalewski, 2007). The climate is subtropical with an average annual temperature of 21.6 ${}^{\circ }$C, with winter temperatures dropping below 0 ${}^{\circ }$C, and an annual average rainfall of 1200 mm (Kowalewski and Zunino, 2004; Kowalewski, 2007).

### 2.2 Data collection

In French Guiana, field observations were carried out from July to September 1993. In Nicaragua, field data were gathered between December 2003 and January 2004 and in February 2006. Finally, in Argentina, data collection occurred in July 2001. By covering only fractions of the years, we acknowledge that it represents a limitation as potential seasonal variation in sleeping habits might not have been captured. All studied howler groups at the three sites were habituated to human observers. To avoid disturbance, monkeys were observed in moonlight whenever possible or by using a flashlight (≤15 lm) without directly focusing on the animals for more than 5 s. All field data were collected using focal sampling before dawn (∼ 04:00 UTC-6: Nicaragua), and after sunset (∼ 19:00 UTC-3: French Guiana and Argentina) when the monkeys had retracted to their sleeping sites, and concerned exclusively adult individuals. Each observed adult individual per night constituted a single sleeping record. Each record involved information on posture, substrate type, substrate diameter, substrate inclination, and the location of each sleeping individual in relation to the vertical and horizontal axis of the tree crown. A total of 143 sleeping records were collected at the three sites (Nicaragua, *A. palliata*, n=55; French Guiana, *A. macconnelli*, n=32; Argentina, *A. caraya*, n=56).

**Figure 2:**
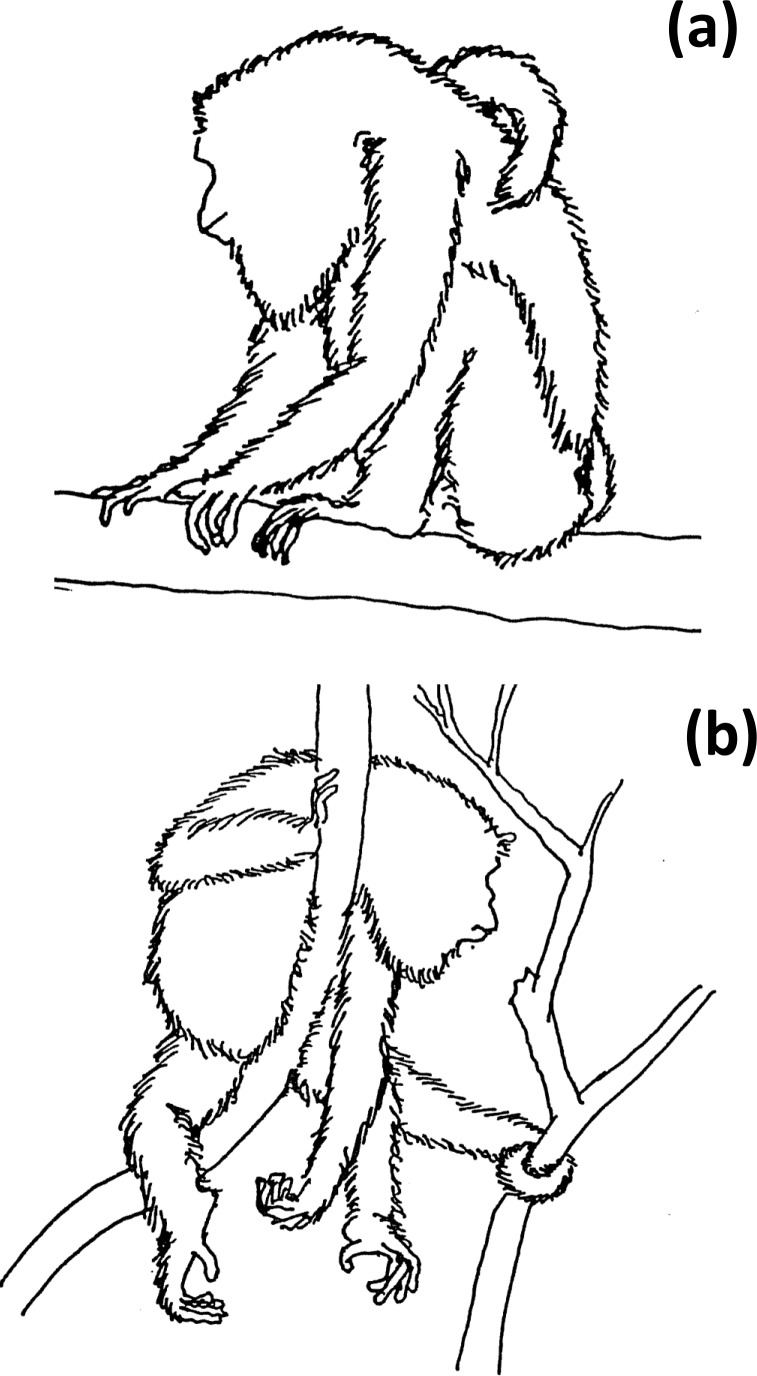
Two sleeping postural modes in *Alouatta*: **(a)** sit-in and **(b)** supine-lie.

Two sleeping postures were identified: *sit-in* and *supine-lie* (Hunt et al., 1996). Sit-in is defined as howlers forming a crouched ball-like posture, with the head, throat, and flexed forelimbs located toward the center of the ventral area of the body, covered by the flexed hindlimbs, while the ischia and feet formed a “tripod” that contacted the supporting tree branch (Fig. 2a). In supine-lie, howlers kept their flexed fore- and hindlimbs under the lying body, placing hands and feet at the same level with the abdominal and inguinal parts of the body, but with at least two fore- and/or hindlimbs, partially hanging (Fig. 2b).

Substrates were defined as “the weight bearing structure on top of which a study subject stands…” (Hunt et al., 1996, p. 366). Substrate type included *unramified* branch and *ramified* branch. When using an unramified branch, monkeys supported their bodies on top of a continuous bough. When employing a ramified branch, howlers placed their bodies on top of the node of a bi- or trifurcation, formed by the main branch and one or two lateral branches. Substrate diameter was based on visual estimation of the branch and was divided into *medium* (5–10 cm) and *large* (>10 cm). No branches <5 cm were recorded. Substrate inclination also involved two categories, in relation to the visually estimated true horizontal: *horizontal* (0–10${}^{\circ }$) and *oblique* (11–30${}^{\circ }$), while no higher inclinations were recorded during sleep.

Regarding canopy location, we considered both vertical and horizontal stratification of tree crowns. Vertically, tree crowns were visually divided into three equal parts: *upper* (top third of the tree crown), *middle* (the central third of the crown), and *lower* (lower third of the crown). Horizontally, the tree crowns were also divided into three sections: *core* (first third of the crown located close to the tree trunk), *middle* (the second and central third of the crown), and *periphery* (the outer third of the crown).

### 2.3 Statistical analyses

Data on frequencies of use of the different variables were calculated. As all our variables were categorical, we used non-parametric statistics. Thus, we apply $G_{\mathrm{adj}}$ and $\chi^{2}$ tests in the analysis of the frequency tables generated by this study (Sokal and Rohlf, 1995; Fowler et al., 1998). We also installed Microsoft™ Excel with the statistical program PopTools to perform these analyses. For the present study, each sleeping focal individual was scanned only once every sleeping night; thus each sleeping individual per night constituted an independent behavioral datum. Moreover, each sleeping night was independent of the next and so were the sleeping focal individuals of each sleeping group each night.

## 3 Results

The sit-in posture was primarily used during sleep, and this pattern was consistent across the three species (Table 1). In terms of substrate inclination, horizontal supports dominated in all three species, with no significant differences across species (Table 1). A similar pattern was detected for substrate size, with all three species using large branches significantly more frequently than medium ones, with no significant differences between them (Table 1). *Alouatta palliata* and *A. caraya* showed no significant differences in substrate type use, using almost equally unramified branches and ramified branches (Table 1). Furthermore, the two species appeared to largely utilize branches with bifurcations (*A. palliata* 75 %, *A. caraya* 96.2 %) over branches with trifurcations. In contrast, *A. macconnelli* used exclusively unramified branches (Table 1).

**Table 1:** Percentages of use of postures, substrate inclination, size and type, and crown location during sleep in three howler monkey species.

	*A. palliata*	*A. macconnelli*	*A. caraya*	$\chi^{2}$
	n=55	n=32	n=56	
Postures				
Sit-in	98.2 %	100.0 %	98.2 %	$0.02^{b}$, n.s.
Supine-lie	1.8 %	0 %	1.8 %	$1.81^{b}$ n.s.
$G_{\mathrm{adj}}$	$65.26^{a}$, p<0.01	–	$66.61^{a}$, p<0.01	
Substrate inclination				
Horizontal	76.4 %	93.7 %	71.4 %	$3.42^{b}$, n.s.
Oblique	23.6 %	6.3 %	28.6 %	$14.12^{b}$, p<0.01
$G_{\mathrm{adj}}$	$15.94^{a}$, p<0.01	$28.94^{a}$, p<0.01	$10.47^{a}$, p<0.01	
Substrate diameter				
Medium	16.4 %	12.5 %	12.5 %	$0.72^{b}$, n.s.
Large	83.6 %	87.5 %	87.5 %	$0.12^{b}$, n.s.
$G_{\mathrm{adj}}$	$26.97^{a}$, p<0.01	$19.94^{a}$, p<0.01	$34.92^{a}$, p<0.01	
Substrate type				
Unramified branch	41.8 %	100.0 %	53.6 %	$29.06^{b}$, p<0.01
Ramified branch	58.2 %	0 %	46.4 %	$54.30^{b}$, p<0.01
$G_{\mathrm{adj}}$	$1.46^{a}$, n.s.	-	$0.28^{a}$, n.s.	
Vertical crown location				
Upper	9.1 %	25.0 %	60.7 %	$44.23^{b}$, p<0.01
Middle	87.3 %	75.0 %	39.3 %	$18.50^{b}$, p<0.01
Lower	3.6 %	0 %	0 %	$7.30^{b}$, p<0.05
$G_{\mathrm{adj}}$	$69.71^{b}$, p<0.01	$8.24^{a}$, p<0.05	$2.56^{a}$, n.s.	
Horizontal crown location				
Core	29.1 %	81.2 %	46.4 %	$27.01^{b}$, p<0.01
Middle	63.6 %	18.8 %	46.4 %	$23.89^{b}$, p<0.01
Periphery	7.3 %	0 %	7.1 %	$7.21^{b}$, p<0.05
$G_{\mathrm{adj}}$	$28.40^{b}$, p<0.05	$13.26^{a}$, p<0.01	$21.91^{b}$, p<0.05	

n.s.: not significantly different. ${}^{a}$ d.f. = 1. ${}^{b}$ d.f. = 2.

Tree crown vertical and horizontal location of sleeping postures across the three species revealed some common patterns. In terms of vertical location, *A. palliata* and *A. macconnelli* largely used the middle parts of tree crowns, while *A. caraya* used both the upper and middle parts (Table 1). All three howler species used the lower part of the tree crown less often. On the other hand, the different howler species preferred the middle and core sections of the trees to a varying extent and appeared to have used the tree periphery less (Table 1).

## 4 Discussion

The current study is a first attempt to systematically record postural behavior, substrate characteristics, and crown location during sleep in three species of howler monkeys across the distributional range of the genus *Alouatta*. Previously, Mendel (1976) provided an early attempt to explore the selection of postures in relation to substrates, tree parts, and anatomical correlations, but his study was restricted to mantled howlers (*A. palliata*). Our comparative study showed that howlers exhibited common sleeping patterns. All three species used the inner parts of tree crowns. Therein, they adopted a sit-in posture on mainly horizontal as well as large, unramified, and ramified branches. Sleep is the major extended behavioral phase of the diel cycle of mammals and appears to play an important role in safety and thermoregulation. The common use of this particular niche (central tree crown with large horizontal branches) by all three species during sleep most likely reflects common adaptive strategies to similar ecological pressures. The central tree crown is a covered zone with reduced access to potential predators and increased protection from extreme weather conditions, and at the same time its abundant large horizontal branches represent steady substrates ideal for postural stability. In this way, they seem to provide the required safety along with mechanical and physiological stability (Anderson, 1984, 1998, 2000).

All three species appeared to have selected parts of the tree crown located in the inner sections, systematically using the periphery and lower part of trees less often. The inner parts of tree crowns usually provide sleeping sites that are relatively inaccessible to potential predators (Zhang, 1995; Anderson, 1998; Ramakrishnan and Coss, 2001). In effect, the central and upper parts of tree crowns provide a natural shelter, reducing visibility to predators, and create a relatively wide buffer zone that may protect animals from approaching predators. In this way, potential howler predators, such as scansorial carnivores (jaguarundis, *Herpailurus yagouaroundi*, jaguars, *Panthera onca*), harpy eagles (*Harpia harpyja*), or humans cannot easily locate prey which is frequently covered in heavy foliage (Peres, 1990; Peetz et al., 1992; Di Fiore, 2002; Kowalewski and Zunino, 2004; Urbani, 2005; Raguet-Schofield, 2008; Urbani et al., 2012). In addition, such locations may also provide protection from extreme weather conditions, as has been suggested for other arboreal monkeys (e.g., Wahungu, 2001; Cui et al., 2006). In our cases, sleeping in central sections of tree crowns may provide cover from heavy and intense rainfalls in French Guiana, from frost during the winter in northern Argentina, and from severe night wind flows in Nicaragua as perceived by the authors in the field sites. However, more detailed data associating these specific factors with canopy part selection are required for further testing.

Apart from a natural hide-out that protects from predators and weather, the inner parts of tree crowns are dominated by relatively large and horizontal branches. Large substrates offer a wide contact area for the body, minimizing any unbalancing forces and the likelihood of toppling over. In effect, all three howler species utilized particularly large substrates, >10 cm in diameter, which is larger than the average width of the pelvis of most howler species (e.g., 7 cm for *Alouatta palliata*: Leutenegger, 1974). In mantled and black-and-gold howlers, the use of branches with bi- and trifurcations further enlarges the contact area for the ischia and enhances stability and balance. In this way, firm and secure postures are guaranteed over an extended time of inactivity during sleep. In addition, all three species mostly used horizontal or only slightly inclined substrates. Horizontal and low-inclination branches assure that the center of mass be located well within the support area, eliminating any risk of sliding, as would have been the case with steeper inclinations. These features characterize biomechanically strong, firm, and stable substrates which provide the stability and equilibrium that is necessary during long postural modes, such as during sleep (Rose, 1974). In this way, animals maintain a dynamic stability with less energy expenditure, thus contributing to energy conservation. This is essential for mammals, such as howlers, that are mainly folivorous and have adopted an overall energy conservation strategy to likely maximize their intake from a low-quality diet (Milton, 1981). Postural stability and equilibrium were further achieved by the principal use of sit-in, as the dominant sleeping posture by all three howler species. Anderson (1984, 2000) has suggested that postural modes such as crouching, sitting, and squatting during sleep in primates appear to provide enhanced stability. These postures enlarge the contact area between body and substrate and simultaneously lower the center of mass of the body, providing balanced support on the branch (Wells, 1974, in Anderson, 2000).

Additionally, crouched sitting postures during sleep have been functionally related to thermoregulation (Anderson, 1984, 2000). Contact between the extremely flexed hindlimbs, forelimbs, and the lowered head towards the ventral area reduces the exposed surface and consequently heat loss (Anderson, 1984, 2000). During stormy weather, the same posture may further divert rainwater away from the body, keeping it warm (Anderson and McGrew, 1984). Regulating heat loss and maintaining a warm body are necessary during sleep, and this is accomplished by adopting this posture within the inner parts of tree crowns with relatively stable microclimatic conditions. The contribution of this posture to thermoregulation is further suggested when night temperatures drop drastically, like in Argentina, where black-and-gold howlers not only use the ball-like sit-in posture but also huddle with other individuals to increase heat retention, as in many other social mammals (Kowalewski and Zunino, 2004; Martín M. Kowalewski, unpublished data; see also Bicca-Marques and Calegaro-Marques, 1998).

Lastly, adopting this crouched ball-like sit-in posture on stable large horizontal branches during sleep for long time periods may be associated with the prolonged digestion times of howlers (Milton, 1981, 1998; Milton and McBee, 1983; Yumoto et al., 1999). It has been suggested that prolonged time of digestion, and the concomitant extended food retention time in the digestive system, aids in the absorption of nutrients in primates (Clauss et al., 2008). Compared to other New World primates, howlers exhibit particularly high rates of folivory (*A. palliata*: Illes, 2005; Raguet-Schofield, 2010; *A. macconnelli*: Julliot and Sabatier, 1993; *A. caraya*: Bravo and Sallenave, 2003; Kowalewski, 2007) and show comparatively long digestion and ingesta retention time than other neotropical primates, which occurs mainly during the night (Milton, 1981; Espinosa-Gómez et al., 2013, Matsuda et al., 2019). Thus, we suggest that the adoption of the sit-in posture during sleep likely provides the necessary stability and positioning for facilitating advanced digestive processes. Similarly, it seems possible that the digestive system is positioned to facilitate the vertical stratification of food particles for digestion and nutrient absorption in hindgut fermentation, over the prolonged nocturnal inactivity and long daily resting periods as commonly observed in howlers. This contention, then, requires further research that might be tackled through extended comparative studies among neotropical primates with different dietary and digestive patterns and gut passage times (e.g., Lambert, 1998). Thus, this ecophysiological mechanism (as observed for foregut fermenters such as wild sloths: Urbani and Bosque, 2007, and colobines: Matsuda et al., 2017) might eventually further interplay with the thermoregulatory and stability contentions summarized by Anderson (1984, 1998, 2000).

The results of the current research have indicated that independent of forest type, howlers tend to use similar substrate features and locations within trees and adopt similar postures during sleep. This common strategy is very likely associated with biomechanical stability, avoidance of predators and/or extreme weather conditions, thermoregulation, the efficiency of the digestive processes, and ultimately phylogeny. These factors may have played an important role in the selection of certain behavioral and habitat variables during sleep. However, in order to further understand these adaptive interrelations, more studies are required on the postural behavior and microhabitat selection during sleep of other howler species and other diurnal neotropical monkeys, as well as other primates with a strong reliance on folivory (Milton, 1998).

## Data Availability

The relevant data of this study are presented in Table 1. Nevertheless, the original dataset is available upon request.
